# Beta-Cell Dysfunction Induced by Tacrolimus: A Way to Explain Type 2 Diabetes?

**DOI:** 10.3390/ijms221910311

**Published:** 2021-09-24

**Authors:** Ana Elena Rodriguez-Rodriguez, Esteban Porrini, Armando Torres

**Affiliations:** 1 Research Unit, Hospital Universitario de Canarias, 38320 La Laguna, Santa Cruz de Tenerife, Spain; anarrguez@gmail.com; 2 Fundación General de la Universidad, Universidad de La Laguna, 38204 La Laguna, Santa Cruz de Tenerife, Spain; 3 Unidad Ensayos Clinicos-UCICEC, Hospital Universitario de Canarias, 38320 La Laguna, Santa Cruz de Tenerife, Spain; atorres@ull.es; 4 Instituto Tecnologías Biomédicas (ITB), Universidad de La Laguna, 38200 La Laguna, Santa Cruz de Tenerife, Spain; 5 Nephrology Department, Hospital Universitario de Canarias, 38320 La Laguna, Santa Cruz de Tenerife, Spain

**Keywords:** pancreatic β-cells, diabetes mellitus type 2, post-transplant diabetes mellitus, tacrolimus, pathways

## Abstract

The combination of insulin resistance and β-cells dysfunction leads to the onset of type-2 diabetes mellitus (T2DM). This process can last for decades, as β-cells are able to compensate the demand for insulin and maintain normoglycemia. Understanding the adaptive capacity of β-cells during this process and the causes of its failure is essential to the limit onset of diabetes. Post-transplant diabetes mellitus (PTDM) is a common and serious disease that affects 30% of renal transplant recipients. With the exception of immunosuppressive therapy, the risk factors for T2D are the same as for PTDM: obesity, dyslipidaemia, insulin resistance and metabolic syndrome. Tacrolimus (TAC) is the immunosuppressant of choice after renal transplantation but it has the highest rates of PTDM. Our group has shown that insulin resistance and glucolipotoxicity, without favouring the appearance of apoptosis, modify key nuclear factors for the maintenance of identity and functionality of β-cells. In this context, TAC accelerates or enhances these changes. Our hypothesis is that the pathways that are affected in the progression from pre-diabetes to diabetes in the general population are the same pathways that are affected by TAC. So, TAC can be considered a tool to study the pathogenesis of T2DM. Here, we review the common pathways of β-cells dysfunction on T2DM and TAC-induced diabetes.

## 1. Introduction

Post-transplant diabetes mellitus (PTDM) affects 20–30% of the patients with a renal transplant [1]. The prevalence, incidence, evolution and consequences of PTDM have been recently reviewed [1]. Diverse risk factors for PTDM have been analysed and described [2]. Clearly, immunosuppressive medications used to avoid organ rejection are the main factor that induces diabetes. Other risk factors for PTDM are obesity, metabolic syndrome, insulin resistance and hypertriglyceridemia [3]. Of note, these factors have been also associated with the incidence of type 2 diabetes in the general population [4]. Our group found that tacrolimus was particularly toxic to β-cells in patients with metabolic syndrome [5]. This was later confirmed in animal models of diabetes induced by TAC [6]. This information led to the hypothesis that TAC may accelerate the same damage in β-cells already induced by insulin resistance and obesity and so help explaining the pathogenesis of diabetes in the general population. Animal and cellular models of glucolipotoxicity seemed to confirm this idea [6,7]. So, understanding tacrolimus-induced β-cells toxicity may help improve our knowledge not only of the pathogenesis of PTDM but also that of type 2 diabetes. In the present review we evaluate common pathways involved in β-cells dysfunction in T2DM and tacrolimus-induced PTDM.

## 2. Physiology of Pancreatic Islets

The pancreatic islets, also called Islets of Langerhans in honour of their discoverer Paul Langerhans in 1869, are irregular structures of about 100–200 μm, formed by accumulations of endocrine cells dispersed among the exocrine acini of the pancreatic tissue [8]. Islets only represent 1–2% of the pancreas and are highly vascularized mini organs receiving five times more blood than the adjacent exocrine tissue [8]. The cytoarchitecture of the islets is complex and varies between species. Islets contain basically four main types of cells. The most abundant are β-cells (50–80%), which secrete insulin, followed by alpha-cells (~20%) that secrete glucagon and PP-cells (10–35%), which secrete pancreatic polypeptide. Delta-cells (~5%) synthetize somatostatin and epsilon cells produce ghrelin and are less frequent [9].

## 3. Pancreatic β-Cell

The β-cell plays a central role in maintaining glucose homeostasis by the production and secretion of insulin. This hormone is a peptide with many diverse functions, including the promotion of glucose and amino acid uptake, glycogen synthase activity, protein metabolism, cell division and growth, as well as decreases lipolysis among the most important [10]. Structurally, insulin has two chains of polypeptides: chain A: 21 amino acids (a.a.), and chain B: 30 a.a., linked by two disulphide bridges. Insulin is synthesized as a precursor, proinsulin, which has a single polypeptide chain made up of the A and B chains and the C-peptide, which joins both chains [11]. The passage from proinsulin to insulin takes place in the trans-Golgi network and needs the breaking of the bonds between the C-peptide and both chains A and, B. Insulin and C-peptide are stored in secretory granules that also contain proteolytic enzymes (convertases), carboxypeptidases, C-peptide, and high concentrations of Ca^2+^. Murine β-cells can have more than 13,000 secretory granules of insulin and each granule may store more than 200,000 insulin molecules [12,13]. The production and release of insulin are highly regulated. The short-term regulation occurs at the release of insulin from granules. Long-term adaptation to glycaemic changes implies the regulation and changes of diverse processes like the transcription of insulin gene, translation of insulin mRNA, processing proinsulin into insulin and stimulation of insulin secretion [12]. The transcriptional regulation of insulin gene involves a sequence of elements within the promoter region of the gene, named, A.; C, E, Z and CRE elements. These are binding sites for important transcription factors that regulate insulin gene expression, such as: v-maf musculoaponeurotic fibrosarcoma oncogene family, protein A (MafA), pancreatic and duodenal homeobox 1 (PDX-1) and β-2/Neurogenic differentiation 1 (NeuroD1) [12].

### 3.1. Mechanisms of Insulin Secretion

**Figure 1:**
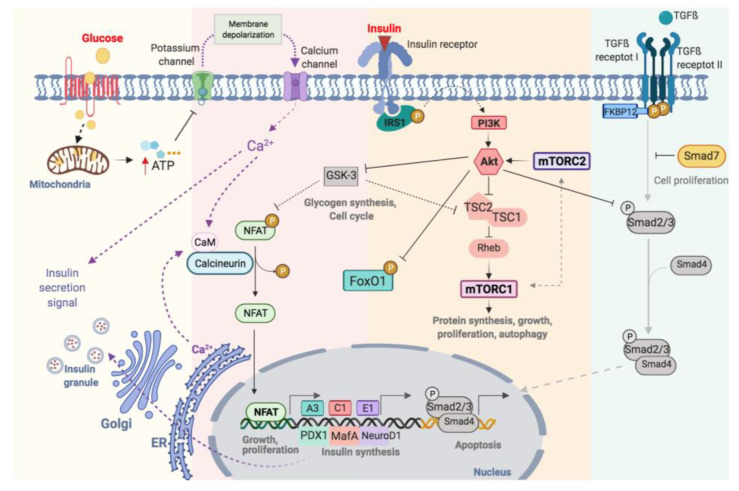
Schematic illustration of functional regulation on pancreatic β-cell.

Glucose is the main stimulator of insulin secretion in β-cells [14]. When extracellular glucose increases, glucose is transported inside β-cells by the glucose transporter (GLUT2) (Figure 1). Once in the cytoplasm, glucose is phosphorylated by glucokinase (GCK) resulting in the formation of glucose-6-phosphate, which is incorporated into the Krebs cycle in the mitochondria increasing cytosolic ATP/ADP ratio (Figure 1). When the β-cell is at rest, the membrane potential remains stable at −70 mV, thanks to the constant flux of K^+^ through ATP-sensitive K^+^-channels (K_ATP_-channels). These channels have two subunits: the pore, Kir6.2, and the regulatory subunit, SUR1 (sulfonylurea receptors) and both assembled with a 4:4 stoichiometry. The increase in ATP, caused by glucose metabolism, favours the closure of K_ATP_-channels. The reduction of K^+^ conductance leads to the depolarization of the cytoplasmic membrane and the opening of voltage-dependent Ca^2+^ channels (VDCCs), which promotes the entry of Ca^2+^ into the cell. This increase in intracellular Ca^2+^ concentration is essential to trigger the fusion of the insulin-containing granules with the membrane and subsequent release of the insulin [15] (Figure 1).

### 3.2. Functional Regulation of β-Cell

Optimal glycaemic control depends on changes in the production and secretion of insulin, as well as the regulation of β-cell proliferation. This process is highly complex and involves many factors, including membrane receptors, intracellular enzymes and proteins, grow factors and hormones (Figure 1). These factors are highly interrelated with a major degree of complexity. Consequently, its understanding requires both comprehensive and simplified approaches. We will describe the most relevant pathways involved in β-cell regulation.

#### 3.2.1. Calcineurin-NFAT Pathway

The increase in intracellular calcium, as a consequence of glucose metabolism, causes the activation of calcineurin, a cytoplasmic calcium-calmodulin-dependent serine/threonine phosphatase, which is responsible for dephosphorylating and translocating NFAT to the nucleus [16] (Figure 1). NFATs are a family of transcription factors that regulate cell proliferation and functional maintenance. Heit et al., demonstrated that the calcineurin-NFAT pathway is important for the maintenance and viability of β-cell. Calcineurin-knockout mice exhibited severe hyperglycaemia with increasing age as well as dysregulation of specific genes like insulin and others implicated in the maintenance of β-cell mass [17]. Glycogen synthase kinase 3 (GSK3) is a serine/threonine kinase implicated in the regulation of glycogen synthesis, protein synthesis and gene transcription [18]. GSK3 regulates NFAT pathway preventing its translocation to the nucleus [19] (Figure 1).

#### 3.2.2. Insulin Receptor-IRS1-PI3K-AKT Pathway

Insulin acts in an autocrine manner promoting β-cell proliferation [20] by the phosphorylation of the insulin receptor substrate 1 (IRS-1) and activation of phosphoinositol-3 kinase (PI3K). This activates Akt, an important serine/threonine kinase involved in multiple processes like the regulation of glucose and insulin metabolism, apoptosis, proliferation and, transcription or cell migration [21] (Figure 1). Fatrai et al. demonstrated that Akt is able to regulate key proteins of the β-cell cycle such as cyclin D1, D2, p21 and increase the activity of cyclin depended kinase-4 (Cdk4) in order to promote β-cell proliferation [22]. The constitutive expression of Akt in β-cells resulted in an increase in cell mass accompanied by hyperinsulinemia [23]. PI3K/Akt phosphorylates several proteins involves in different processes such as proliferation, cell growth, differentiation and survival (Figure 1). Akt phosphorylates the tuberous sclerosis complex (TSC1/TSC2) inhibiting its GTPase activity allowing Rheb actives mTOR [24]. Thus, Akt and mTOR are closely related. mTOR can be part of two complexes: the mammalian target of rapamycin complex 1 (mTORC1) and 2 (mTORC2). For the complete activation of Akt, mTORC2 phosphorylates Akt in Ser-473 [24]. Akt regulates the cell cycle through the phosphorylation and inhibition of GSK3. The latter is related to the mTORC1 pathway by phosphorylating and inhibiting TSC2 [25,26]. Thus, the activation of Akt, via insulin, inactivates GSK3 and stimulates proliferation via mTORC1 (Figure 1). Also, Akt binds to the transcription factor Smad3 and prevents its binding to Smad4 and translocation to the nucleus for apoptosis induction (Figure 1) [27].

#### 3.2.3. mTOR Pathway

mTOR is a key serine/threonine kinase that controls multiplex cellular processes in response to a variety of environmental stimuli including amino acids, glucose and oxidative stress [28,29] (Figure 1). As we commented above, mTOR is a protein can be part of two different complex: mTORC1 and mTORC2 [24]. The mTORC1 phosphorylates S6 kinase (S6K) and eukaryotic initiation factor 4E-binding protein-1 (4EBP1) [29]. The activation of this pathway stimulates ribosomal biogenesis and mRNA translation promoting the biosynthesis of protein. mTORC1 also regulates lipid biosynthesis by activating the major lipogenic regulator transcription factor SREBP1, which controls the expression of numerous genes involved in fatty acid and cholesterol synthesis [30].

#### 3.2.4. Nuclear Factors Relevant in β-Cells Metabolism: PDX1, MafA, NeuroD and FoxO1

PDX-1 is considered the main transcription factor involved in the early development of the pancreas, β-cells differentiation and maintenance of β-cells mass. PDX-1 regulates transcription of genes such as insulin, GLUT2, glucokinase, NKx6.1 and MafA, among others [31,32,33]. Mice with a selective disruption of the Pdx1 gene in β-cells develop diabetes associated with a reduction in insulin production and GLUT2 expression. Mice heterozygous for Pdx1 develop glucose intolerance, increased apoptosis, decreased mass and abnormal islet architecture. This indicated that PDX-1 is crucial for maintaining the regulation of glucose metabolism [33]. In humans, mutations in this gene cause a monogenic form of type 2 diabetes, known as MODY 4 (maturity -onset diabetes of the young 4) [34].

MafA is a critical transcription factor for the maintenance of mature β cells [35]. It is only expressed in β-cells and acts as a potent activator of the insulin gene. Knockout mice for MafA develop glucose intolerance and diabetes, lower expression of the insulin gene, PDX-1, NeuroD and GLUT2 [36,37,38].

NeuroD1 is involved in pancreatic development and endocrine cell differentiation. It is expressed in mature β cells and, together with PDX1 and MafA, binds directly to the promoter of the insulin gene and controls its transcription [39].

Forkhead box protein O1 (FoxO1) is an important transcription factor with a central role in multiple processes such as differentiation, proliferation, apoptosis, cellular metabolism and response to cellular stress [40,41,42,43,44]. In β-cells, FoxO1 is a key transcription factor in the regulation of insulin and glucose homeostasis in response to stress. It is interesting to note that the levels of mRNA-foxo1 are higher in diabetic patients [45]. The activation of Akt via PI3K leads to the nuclear exclusion of FoxO1 (Figure 1) [46]. FoxO1 also regulates the expression of PDX-1 by two possible mechanisms: (1) the control the subcellular localization of PDX1, presenting an exclusive pattern within the nucleus, and (2) repressing the expression of FOXA2 (forkhead box protein A2), which in turn controls the promoter of Pdx1 [47]. Therefore, the regulation of PDX-1, MafA, NeuroD and FoxO1 is key to maintaining the functionality of β-cells [40].

#### 3.2.5. TGF-β Receptor Pathway

Transforming growth factor β (TGF-β) superfamily includes TGF-β, activins and BMPs (bone morphogenetic proteins) regulates multiple cellular processes like proliferation, diffentiacion and apoptosis [48]. The activation of this pathway requires the phosphorylation of Type II and I transmembrane protein serine/threonine kinases receptors (TβR-II and TβR-I). Then, TβR-I phosphorylates Smad2 and Smad3 (R-Smads) and forms a complex with Smad4 (Co-Smads). The complex translocates from the cytoplasm to the nucleus and regulates the transcription of genes. In β-cells, the activation of TGF-β/Smad3 represses insulin transcription reducing insulin content and secretion. Also, reduces the expression of the majority genes involved in β-cells function such as SUR1, FoxO1, Ins-1, Ki6.2, PDX-1, NeuroD, Nkx6.1 [49]. The inmunophilin FKBP12 binds to TβR-I and inhibits its phosphorylation by TβR-II, preventing the uncontrolled activation of TGFβ receptor [50,51]. The inhibitory Smads (I-Smad), like Smad7, negatively regulates the TGFβ pathway by the ubiquitination and degradation of TβRs and R-Smads or a simple disruptor of interactions between R-Smads and Co-Smads or TβRs and R-Smads [52] (Figure 1).

## 4. Dysfunction of β-Cell: Insulin Resistance, Glucolipotoxicity and Diabetes

The combination of insulin resistance and pancreatic β-cell dysfunction plays an important role in the pathogenesis of type-2 diabetes mellitus (T2DM). During the progression to T2DM, β-cells go through different changes in mass, phenotype and function. Insulin resistance is accompanied by hyperglycaemia and hyperlipidaemia and increased insulin secretion to maintain normal glucose levels. This compensatory phase may last for decades but in the long term may lead to β-cell dysfunction and loss. In fact, in T2DM, β-cell mass may be reduced up of 60% [53]. Although not fully understood, several mechanisms have been proposed to explain the loss of β-cell mass and function, including apoptosis (classical theory) [53,54] and dedifferentiation (new theory) [55,56,57,58,59]. Obesity associated to IR causes an increase in free fatty acids (FFA), glycaemia and pro-inflammatory mediators, such as adipokines and cytokines [60]. This status of glucolipotoxicity promotes oxidative stress and damages relevant cellular structures like the mitochondria and endoplasmic reticulum, leading to a reduction in insulin synthesis and secretion [60,61]. Glucolipotoxocity affects the main pathways related to the maintenance of β-cell functionality as described above.

### 4.1. Glucolipotoxicity and the Calcineurin-NFAT Pathway

**Figure 2:**
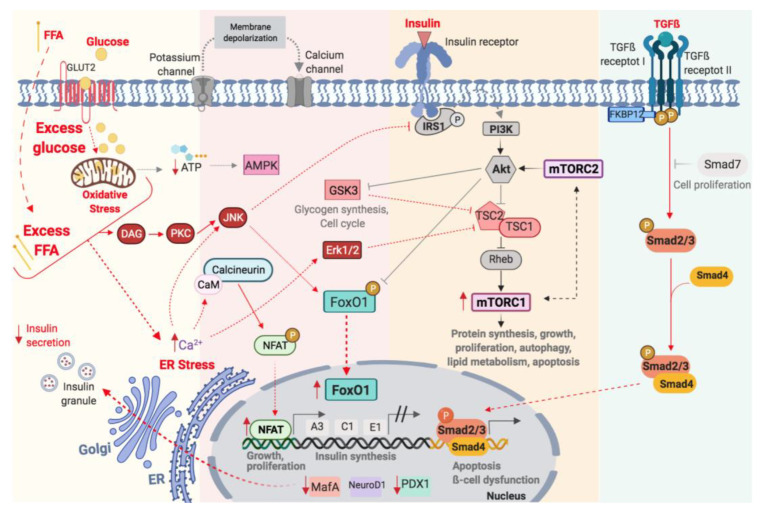
Schematic illustration of the activation of molecular pathway by glucolipotoxicity on pancreatic β-cell.

The exposure to glucose and fatty acids cause an increase in free cytosolic Ca^2+^ concentration, calcineurin activity and expression of nuclear NFAT [7] (Figure 2). Importantly, glucose-stimulated insulin secretion is triggered by an increase in free cytosolic Ca^2+^ concentration [62], but in chronic glucolipotoxic conditions, calcium dynamics are dysregulated due to ER stress [63] and decreased mitochondrial activity, insulin gene expression, insulin content, insulin granule docking to the membrane and insulin secretion [64]. Thus, the maintenance of normal Ca^2+^ concentration is critical to β-cell survival [62]. The reduction in mitochondrial activity diminishes glucose-stimulated ATP generation. AMP-activated protein kinase (AMPK) is a key regulator of intracellular energy balance, acting to maintain ATP levels in response to reduced energy availability (Figure 2). In β-cells, the activation of AMPK reduces the ability to secrete insulin in response to glucose [65,66]. Metformin, the most widely used drug for T2DM, directly stimulates AMPK and the latter inhibits mTORC1 activity by directly phosphorylation of TSC2 or Raptor (an essential component of mTORC1), suppressing protein synthesis and β-cell proliferation [67,68].

### 4.2. Gluocolipotoxicity and the Insulin Receptor-IRS1-PI3K-AKT Pathway

The toxic effect of glucose and lipids increases oxidative stress and production of reactive oxygen species (ROS) reducing glucose-stimulated ATP generation and promoting ER stress. Altogether these changes lead to the activation of the c-Jun N-terminal kinase (JNK) pathway [69] (Figure 2). The JNK modulates a diverse number of cellular processes, including apoptosis, proliferation, inflammation and metabolism. JNK phosphorylates the serine residue of IRS-1 which inhibits the insulin receptor-IRS-PI3K-Akt pathway [70]. This process determines a resistance to the action of insulin (Figure 2). Decreased Akt activity, as a result of IRS-1 phosphorylation by JNK, leads to a decrease in the phosphorylation of FoxO1 promoting its nuclear translocation (Figure 2). Moreover, JNK directly phosphorylates FoxO1 inducing its translocation to the nucleus [70]. Thus, JNK can active FoxO via direct and indirect mechanism. Nuclear FoxO1 affects the intracellular localization of PDX-1 and in consequence, in the maintaining of normal β-cell function (Figure 2).

### 4.3. Glucolipotoxicity and the mTOR Pathway

Glucose, in the presence of FFA, results in synergistic effects on ER stress, impaired insulin receptor-PI3K-Akt signalling and GSK3 activation [71] (Figure 2). GSK-3 is a serine/threonine kinase implicated in the regulation of glycogen synthesis, protein synthesis and gene transcription. In the context of glucolipotoxicity, where the PI3K-Akt pathway is decreased, the expression and activity of GSK-3 increases [72] and is able to mediate mTOR activation [25]. This activation leads to β-cell hypertrophy and the state of hyperinsulinemia that characterizes insulin resistance [26,73]. In β-cells, the serine/threonine kinases, extracellular signal-related kinase 1 and 2 (ERK1/2) are activated by insulin, growth factors and nutrients. Their activation depends on the need to maintain normoglucemia [74]. ERK1/2 are able to promotes the activation of mTOR pathway by inhibiting the TSC1/TSC2 complex [75,76]. Thus, ERK1/2 and PI3K pathway cooperate to activate mTOR and promote cell proliferation (Figure 2). It important to know that chronic or inappropriate hyper-activation of mTOR can lead to β-cell failure and is a common feature of many diseases, including obesity and T2DM [29,77].

### 4.4. Glucolipotoxicity and the Nuclear Factors: PDX, MafA, NeuroD and FoxO1

The prolonged exposure to glucose and FFA causes the gradual loss of β-cell specific genes like PDX-1, MafA, NeuroD and FoxO1, that provide β-cell functionality and identity [40]. As mentioned above, the increase of the ROS and oxidative stress, as a consequence of glucolipotoxicity, active JNK and ERK pathways that suppress the binding of PDX-1, MafA and NeuroD1 to the insulin promoter and decrease insulin gene expression [78,79] (Figure 2). Moreover, in these conditions, the increase in a expression of nuclear FoxO1 is able to block the β-cell proliferation by downregulation of PDX-1, due to exclusive patterns of nuclear localization [40]. The loss of MafA in β-cell leads to a deeper loss of cell identity, which is implicated in diabetes pathology [35]. The dedifferentiation or transdifferentiacion has been proposed as a mechanism of loss of β-cell in diabetes. Talchai et al. [55], in a mouse model lacking of FoxO1 in β-cell, demonstrated that the progressive disappearance of PDX1 and MafA led to the appearance of typical markers of undifferentiated cell such as Neurogenic-3 od Oct-4. Wang et. al. [80], in a model of K_ATP_-dependent diabetes, demonstrated that the dedifferentiation, rather than apoptosis, was the main mechanism for loss of β cells, and the insulin therapy caused redifferentiation to mature β cells. Thus, in addition of apoptosis, the dedifferentiation could be characterized by the decrease in expression of these key β-cell genes as a mechanism of protection from on-going stress [59].

### 4.5. Glucolipotoxicity and the TGF-β Receptor Pathway

The expression of TGF-β is increased in diabetes and obesity [81]. The glucolipotoxicity enhances oxidative stress and the expression of TGF-β [82]. Moreover, the decrease Akt signalling, due to IR and glucolipotoxicity, also results in a direct activation of Smad3 and stimulate apoptosis [83] (Figure 2). In β-cell, Smad3 represses insulin promoter activity and regulates the expression of genes involved in β-cell function like Insulin 1 and 2, Glut2, Glucokinase, SUR1, FoxO1, Ki6.2, Snap25, PDX-1, NeuroD1 and NKx6.1 [49]. Thus, the activation of Smad3 promotes apoptosis and β-cell dysfunction, glucose intolerance and diabetes [84].

## 5. Post-Transplant Diabetes Mellitus (PTDM) and Tacrolimus

PTDM is a complex and multifactorial disease that in several aspects resembles T2DM. Several risk factors for PTDM have been described [85]. In brief, the immunosuppressive therapy used in renal transplanted patients is the major determinant of PTDM and other risk factors include obesity, metabolic syndrome, insulin resistance, and hypertriglyceridemia. Previous publications showed that metabolic syndrome in patients in the waiting list are those at major risk for PTDM, particularly when tacrolimus is given to avoid rejection [85]. The high prevalence of metabolic syndrome and obesity in the waiting list for transplantation and the spread use of tacrolimus may explain the high prevalence of PTDM in renal transplant patients [85]. This group of metabolic factors have been frequently associated in the general population with an increased incidence of T2DM, an issue that indicates common roots between PTDM and T2DM. Of interest, recent studies observed that tacrolimus, the most commonly used immunosuppressive, affects the same pathways in β-cells than obesity and metabolic syndrome. So, understanding β-cell damage by tacrolimus may help clarifying the view of β-cell dysfunction in T2DM. In this section we will describe evidence on common causes of cell damage with TAC.

### 5.1. Common Actions between Tacrolimus and Glucolipotoxicity

**Figure 3:**
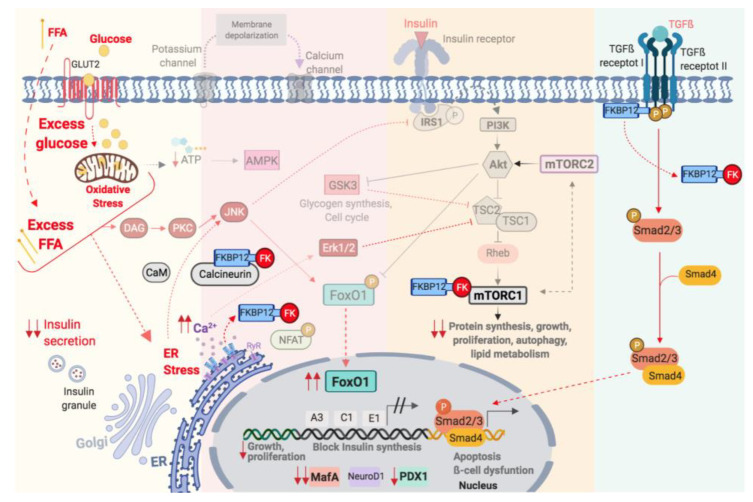
Schematic illustration of the action of Tacrolimus in the context of glucolipotoxicity on pancreatic β-cell.

Tacrolimus (TAC) is a macrolide extracted from a *Streptomyces* *tsukubaensis* [86] that acts as a potent immunosuppressive agent. TAC, together with cyclosporine A (CsA), constitutes the so-called calcineurin inhibitors (CNIs). The mechanism of action is similar for both CNIs. TAC and CsA bind with high affinity and specificity to their cytoplasmic receptors: FKBP12 and Cyclophilin, A.; respectively, to inhibit calcineurin (Figure 3). Calcineurin is a calcium-calmodulin-dependent serine/threonine phosphatase, which dephosphorylates the nuclear factor NFAT allowing its translocation to the nucleus [87,88]. This transcription factor causes the activation of several genes, including IL-2, responsible for regulating the correct immune response. The immunosuppressive capacity of TAC and CsA has been widely demonstrated [89,90]. However, the effect of both medicaments on β-cells is poorly understood.

### 5.2. Calcienurin-NFAT Pathway Inhibition Contributes Diabetogenic Effect of TAC and CsA

The calcineurin-NFAT pathway was the first signalling studied in β-cell damage by CNIs. In 2006, Heit et al. [17] created mice with a specific inactivation of the β-cell calcineurin subunit (βCnb1KO), and these mice presented severe hyperglycaemia with increasing age, leading to diabetes. The complete inactivation of this pathway caused deregulation in the insulin gene, as well as those involved in the proliferation and maintenance of the β-cell mass. They concluded that the calcineurin-NFAT pathway is important for the maintenance and viability of the pancreatic β cell [17]. Therefore, it is possible to speculate that inhibition of this pathway, mediated by the use of CNIs (TAC or CsA), could contribute to the diabetegenic effect of CNIs.

### 5.3. TAC and PTDM: Beyond the Inhibition of Calcienurin-NFAT Pathway

The inhibition of calcineurin-NFAT pathway has been proposed for decades as a main mechanism for PTDM induced by TAC or CsA [17]. Moreover, as TAC is a more potent inhibitor of calcineurin than CsA [91,92], this has been proposed as the cause of the higher diabetogenic effect of TAC compared with CsA. However, this higher inhibition of calcineurin by TAC has been found originally in T-lymphocytes [7,90,91,92] but has not been confirmed in β-cell. Few studies have analysed inhibition of the calcineurin–NFAT pathway in β-cell. In 2011, Ozbay et al. [93] analysed this effect of CNIs on β-cell and observed that TAC and CsA inhibited, to the same extent, calcineurin and glucose-stimulated insulin secretion in INS-1 cells. In line with this study, our group has evaluated the effect of TAC and CsA on β-cell in an in vivo model of obesity and insulin resistance: obese (fa/fa) and lean (fa/-) Zucker rats [6], and in an in vitro model of metabolic stress: INS-1 cells treated with glucose and palmitate [7]. In a in vivo model, we observed that TAC induced diabetes in 100% obese animals and only 40% on CsA. In the lean animals either TAC or CsA developed diabetes. Moreover, TAC in obese animals reduced β-cell proliferation and *Ins2* gene expression compared with CsA but the nuclear NFAT expression was similar between TAC and CsA [6]. In an in vitro model, the treatment with metabolic stressors, glucose and palmitate, increased the activity of calcineurin and expression of NFAT [7]. This is in line with previous studies showing that calcineurin and NFAT are active in the context of hyperglycaemia [94,95]. In addition to this overactivation with metabolic stressors, both TAC and CsA reduced, in the same way, calcineurin activity and NFAT expression [7]. Therefore, the calcineurin-NFAT pathway contributes to the diabetogenic effect of CNIs but is not the cause of the higher diabetogenic effect of TAC compared to CsA. Also, not all studies observed that TAC was more diabetogenic than CsA. Porrini et al. [5], in a retrospective study of 314 patients found that the incidence of PTDM in patients on TAC or CsA was comparable. However, TAC was more diabetogenic than CsA only when given to patients with metabolic syndrome [5]. In our in vivo [6] and in vitro [7] studies, commented above, we confirmed this hypothesis. Only in obese animals and in INS-1 cells treated with glucose and palmitate, TAC was more diabetogenic than CsA. Thus, the interaction between TAC and insulin resistance or glucolipotoxicity may explain, in part, the different diabetogenic effect between CNIs. Thus, in addition to the common calcineurin-NFAT pathway, TAC may affect more pathways in β-cell than CsA.

### 5.4. TAC Accelerates an On-Going Process: Insulin Receptor-IRS1-PI3K-Akt Pathway

Glucolipotoxicity activates JNK in β-cell resulting in the phosphorylation of IRS-1 impairment of insulin-Akt signalling and reduction of insulin gene transcription [69]. In in vivo [6] and in vitro models [7], TAC potentiated the effect of glucolipotoxicity: decreasing the Akt phosphorylation (Rodríguez-Rodríguez, A.E., personal communication), and reduced β-cells proliferation and insulin gene expression [6]. Soleimanpour et al., observed similar results: a reduction of Akt phosphorylation [96] and phospho-GSK-3, a substrate of Akt, with TAC [96] Also, TAC contributes to the reduction β-cells proliferation by its interaction with the mTOR pathway [25]. Therefore, TAC favours or potentiates the damage in the β-cell proliferation induced by the glucolipotoxicity state (Figure 3).

### 5.5. TAC Accelerates an On-Going Process: mTOR Pathway

mTORC1 is an important regulator of cell proliferation and cellular stress. In the context of glucolipotoxicity, mTORC1 increased the activity to compensate the excess of nutrients [97,98]. In this context of overactivation, a study of our group showed that TAC inhibited the mTOR pathway as reflected by lower levels of phospho-mTOR, and two downstream effectors: phospo-p70S6K, and phospo-S6 [98]. How TAC inhibits the mTOR pathway is not clear, but an interaction between TAC-FKBP12 and mTOR may be possible as indicated by in silico docking and inmunoprecipitation analysis [98] (Figure 3).

### 5.6. TAC Accelerates an On-Going Process: PDX-1, MafA, NeuroD and FoxO1

In the β-cell, prolonged exposure to glucose and FFA causes a gradual loss of key transcription factors [41]. Mouse model of obesity and insulin resistance showed a reduction in nuclear MafA [41,99]. The nuclear exclusion of FoxO1 is necessary for β-cell proliferation in the insulin resistance state [43,100]), but under these conditions, a decrease in Akt activity alleviates the inhibition of *FoxO1*, which translocates to the nucleus and represses its target genes like *PDX-1* [101]. Thus, glucolipotoxicity favors the loss of key transcription factors for the β-cell functionality: PDX-1, MafA and NeuroD [41,102] (Figure 3). In line with these studies, TAC potentiates these same changes of metabolic stress causing a drastic reduction in nuclear MafA and an increase in nuclear FoxO1 only when TAC was administrated in combination with metabolic stressor [7]. Interestingly, in the absence of stressors (glucose and palmitate), TAC did not induce these changes. Consequently, TAC must share similar pathways in the induction of β-cell dysfunction that insulin resistance and obesity milieu.

### 5.7. TAC Accelerates and On-Going Process: TGF-β Receptor Pathway and Intracellular Calcium

In this point, it is important to remember that TAC bind to FKBP12 to exert it is biological actions. FKBP12 is an ubiquitous and abundant protein that act a cytoplasmic receptor for TAC which is able to regulate, on the one hand, the TGF-β/activin/BMP superfamily of receptors: FKBP12 binds to receptor I (Type I receptor), stabilizing the conformation inactive and blocking access to substrates [51,103,104,105]. Thus, TAC is able to compete with the Type I receptor to bind to FKBP12. The release of FKBP12 from the receptor in absence of substrate could favour the activation of Smads pathway (Figure 3). In line with this, Triñanes et al., in an in vitro model of primary human islets and in an in vivo model of human islets transplanted into the high fat diet mice, showed that TAC induced β-cells failure with decrease in MafA expression, through activation of BMP/Smad pathway only when TAC was administrated under metabolic stress conditions [105]. On the other hand, FKBP12 is able to regulate Ca^2+^ channel ryanodine receptors (RyRs): each RyR homotetramer binds to FKBP12 and stabilizes channel closure and prevents uncontrolled leakage of Ca^2+^ from the endoplasmic reticulum [106]. Therefore, TAC is able to compete with RyR for FKBP12 and deregulates the intracellular calcium mobilization, enhancing the effect of glucolipotoxicity (Figure 3). Thus, pathways related to FKBP12 could be modified by TAC and associated with the diabetogenic effect of TAC in the context of glucolipotoxicity. This point deserves proper attention in futures studies.

## 6. Conclusions

The factors involved in β-cell damage in the evolution from metabolic syndrome-prediabetes to T2DM are multiple. These factors are highly interrelated with a major degree of complexity and involve multiples pathways responsible of β-cells maintenance and function. These pathways are insulin/PI3K/Akt, mTOR, Calcineurin/NFAT, TGF-β receptor and nuclear factors. TAC acts in most of these pathways accelerating the previous damage induced by glucolipotoxicity. Thus, TAC may serve as a catalytic effector of β-cell damage in the context of obesity and metabolic syndrome. So, TAC may help in the study of the pathways involved in β-cell damage in development of diabetes. This utility of TAC deserves be the focus of attention of future studies.
